# Association between femur size and a focal defect of the superior femoral neck

**DOI:** 10.1016/j.bone.2015.06.024

**Published:** 2015-12

**Authors:** A.H. Gee, G.M. Treece, C.J. Tonkin, D.M. Black, K.E.S. Poole

**Affiliations:** a University of Cambridge Department of Engineering, Trumpington Street, Cambridge CB2 1PZ, UK; b University of Cambridge Department of Medicine, Level 5, Addenbrooke's Hospital (Box 157), Hills Road, Cambridge CB2 2QQ, UK; c University of California, San Francisco, Department of Epidemiology and Biostatistics, 185 Berry Street, Lobby 5, Suite 5700, San Francisco, CA 94107, USA

**Keywords:** Cortical bone mapping, Osteoporotic fractures, Hip structure analysis

## Abstract

Within each sex, there is an association between hip fracture risk and the size of the proximal femur, with larger femurs apparently more susceptible to fracture. Here, we investigate whether the thickness and density of the femoral cortex play a role in this association: might larger femurs harbour focal, cortical defects? To answer this question, we used cortical bone mapping to measure the distribution of cortical mass surface density (CMSD, mg/cm^2^) in cohorts of 308 males and 125 females. Principal component analysis of the various femoral surfaces led to a measure of size that is linearly independent from shape. After mapping the data onto a canonical femur surface, we used statistical parametric mapping to identify any regions where CMSD depends on size, allowing for other confounding covariates including shape. Our principal finding was a focal patch on the superior femoral neck, where CMSD is reduced by around 1% for each 1% increase in proximal-distal size (*p* < 0.000005 in the males, *p* < 0.001 in the females). This finding appears to be consistent with models of functional adaptation, and may help with the design of interventional strategies for reducing fracture risk.

## Highlights

• We analysed QCT scans of the proximal femurs of 308 males and 125 females.
• We mapped the cortical mass surface density (CMSD) across each proximal femur.
• In a focal patch on the superior femoral neck, CMSD decreases as femur size increases.
• This finding is consistent with models of functional adaptation and fracture risk.

## 1 Introduction

The relationship between hip fracture, bone strength and the geometry of the proximal femur has been much studied but poorly understood. For the sake of concision in this short paper, we cite primarily the review by Gregory and Aspden [7], to which the reader may refer for an extensive bibliography. Traditionally, hip geometry has been assessed in DXA images or plain radiographs using intuitive measures such as hip axis length, femoral neck axis length, femoral neck width and neck-shaft angle. Much of the literature examining these measures appears contradictory. For example, Gregory and Aspden [7] identify six studies that found a larger femoral neck width in both male and female fracture cases, but three studies that found the opposite. Rivadeneira et al. [16] concur, stating that “the relation between femoral neck width and the risk of fracture remains conflicting; whereas some find a smaller neck width a risk factor, we and others find a wider neck as risk factor for hip fracture.” There is a greater consensus for hip axis length and femoral neck axis length, with most studies finding a positive relationship between longer lengths and fracture risk, but some finding no significant association [7]. Of the principal measures of hip geometry, only the effect of neck-shaft angle appears to be completely uncontentious, with larger angles associated with increased fracture risk, particularly for trans-cervical fractures, and reduced bone strength [7]. These observations are just as valid today as they were in 2008, with Machado et al. [10] making much the same points in the introduction to their recent paper.

Gregory and Aspden [7] suggest that many of the apparent contradictions can be attributed to inconsistent nomenclature, measurement techniques and outcome measures. They go on to argue that examining geometrical measures in combination, rather than in isolation, is particularly problematic, since the various measures are often correlated with each other. It follows that the outputs of models predicting fracture risk, where the models are based on correlated regressors, need interpreting with great care. The use of different models, incorporating different types and numbers of regressors, might further explain some of the apparent contradictions.

Gregory and Aspden [7] make a compelling case for a more “holistic approach”, by which “shape” is decoupled from “size” and parameterized along orthogonal vectors derived from principal component analysis of the population. In other words, the size and shape of a femur should be expressed using a small number of linearly independent parameters, which between them capture most of the population variance, while avoiding the complications of correlated measures. This is exactly the approach we take here, using 3D shape modelling to describe the proximal femur in terms of linearly independent “size” and “shape” parameters. We focus attention on the single “size” parameter, since it alone accounts for nearly 60% of the population variance. Our specific objective is to test whether, and if so precisely where on the proximal femur, a femur's “size” affects the thickness and density of its cortex. To answer these questions we apply the recently developed technique of cortical bone mapping [19], [20], [21].

## 2 Methods

### 2.1 Study design

The *Osteoporotic Fractures in Men* (MrOS) study recruited 5994 men in the USA between March 2000 and April 2002 [3], [12]. Eligible subjects from six clinical sites were 65 years of age or older, able to walk without assistance, and had not had bilateral hip replacement surgery. A randomly selected cohort of 308 individuals, all with baseline QCT scans, constitutes the male subjects in the present work. The QCT scans were performed on a variety of machines, all including a calibration phantom (three-compartment, Image Analysis Inc., Columbia, KY, USA) for converting from Hounsfield Units to bone mineral density. A statistical analysis plan was submitted to the MrOS Publications Committee before receipt of the demographic data.

The female subjects were drawn from two retrospective case-control studies of hip fracture in women. The *Regional Thinning of the Femoral Neck Cortex in Hip Fracture* (FEMCO) study recruited 161 women in the UK, 50 of whom were healthy volunteers attending Addenbrooke's Hospital, Cambridge. The *Study of Hip Joint in Trauma* recruited 150 women in the Czech Republic, 75 of whom were healthy volunteers attending Homolka Hospital, Prague. The QCT scans were performed on a variety of machines, all including a calibration phantom (five-compartment, Mindways Inc., Austin, TX, USA at Cambridge; two-compartment, Siemens AG, Erlangen, Germany at Prague). The female subjects in the present work comprise the 50 UK and 75 Czech controls, producing a sample size of 125. There was no a priori intention to examine this data in this study. Rather, for reasons that will be discussed in Section 4, there arose a need to validate the MrOS results, with the ancillary benefit of extending the conclusions to females. The FEMCO and Prague data was readily available to the authors, having previously been analysed in fracture case–control studies, and must therefore be viewed as a convenience sample.

Demographics for the male and female subjects can be found in Table 1. Informed consent was obtained from all participants.

### 2.2 Cortical bone mapping

**Fig. 1:**
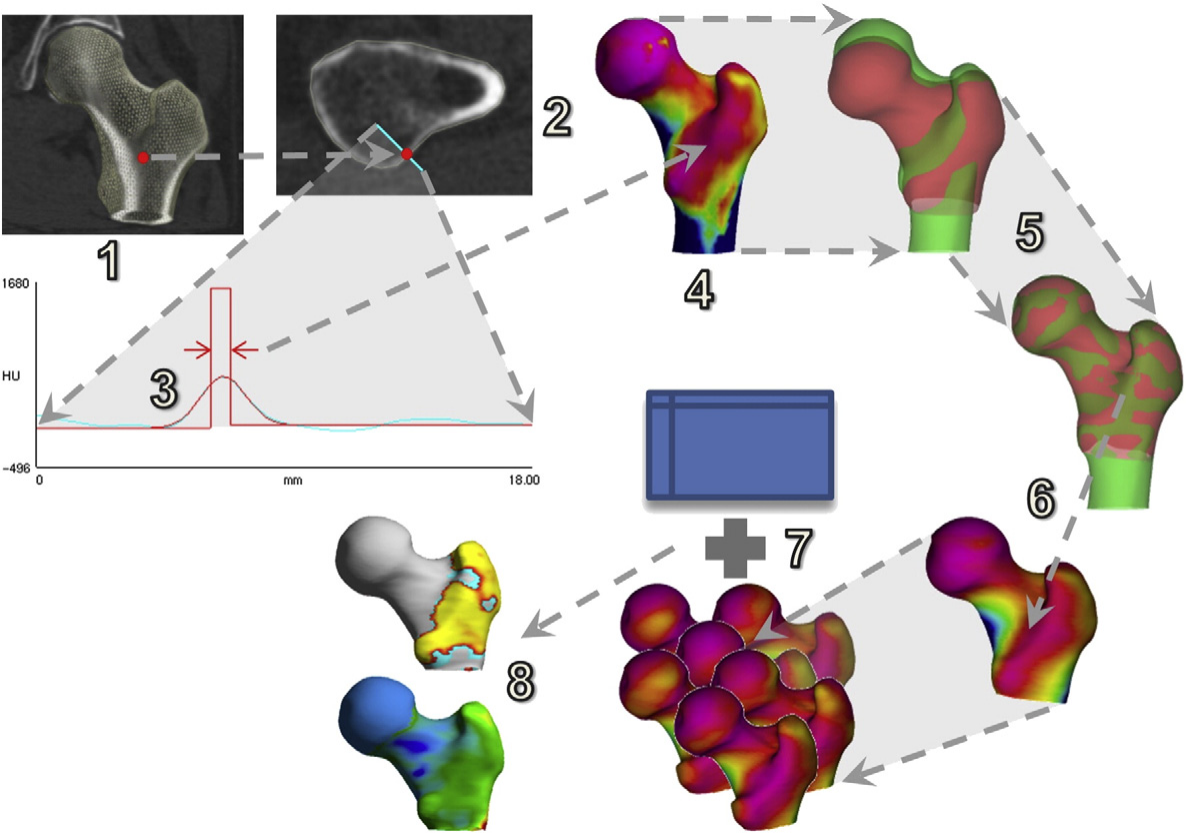
Cortical bone mapping (1–4), spatial registration (5–6) and statistical parametric mapping (7–8).

Cortical bone mapping [19], [20], [21] is a novel technique that estimates the cortical thickness (CTh, cm), cortical bone mineral density (CBMD, mg/cm^3^) and cortical mass surface density (CMSD = CTh × CBMD, mg/cm^2^) at thousands of locations distributed over the proximal femoral surface. An overview of the process can be found in Fig. 1. The starting point is an approximate segmentation of the proximal femur, represented by a triangular mesh with ~ 10^4^ vertices (Fig. 1, step 1). At each vertex, the CT data is sampled along a line passing perpendicularly through the cortex (step 2). A model (step 3, red straight lines), that accounts for the imaging blur, is fitted to the data (step 3, cyan curve) so as to minimize the differences between the blurred model (step 3, red curve) and the data. This is repeated at all vertices: the resulting distributions of CTh, CBMD and CMSD can be visualised as colour maps on the femoral surface (in step 4, pink is low CMSD while blue is high CMSD). Software to perform the initial segmentation and the cortical bone mapping is available for free download.^1^

The resolvability of cortices in CT images depends on *σ*, the standard deviation of the assumed Gaussian imaging blur. For thick cortices (thickness greater than 4*σ*, or around 3 mm for typical clinical resolution), CBMD and CTh can be resolved unambiguously, since the cortex is sufficiently thick for its actual density to be apparent in the CT data. For thinner cortices, the model-fitting process (Fig. 1, step 3) becomes increasingly ill-posed, since a dense, thin, blurred cortex looks very similar to a less dense, less thin, blurred cortex. CMSD, the product of thickness and density, is unaffected by this ambiguity and remains estimable by a variety of techniques [19], [21], but decomposing CMSD into CTh and CBMD is more difficult. The approach taken in [19], and adopted here, is to initially estimate single, global values of CBMD and *σ* across the entire proximal femur. The global CBMD estimate is then used to constrain the model-fitting process at each vertex, yielding local values of CTh and *σ*. Finally, the discrepancy between the local and global values of *σ* is used to adjust the per-vertex CBMD and CTh estimates. This method achieves CTh accuracy of 0.12 ± 0.39 mm for cortices in the range 1–3 mm [19]. Unsurprisingly, given the thickness-density ambiguity, the most accurate and precise estimates are for CMSD [19], which is one of the reasons why we focus on this parameter in the present work. The other reason is that it is likely to play a significant role in local fracture resistance, accounting as it does for both the amount of cortex (CTh) and the mineralization of said cortex (CBMD).

### 2.3 Statistical methods

For a cohort of size *n*, cortical bone mapping results in *n* spatial distributions like the one in Fig. 1, step 4, each expressed on a different triangular mesh (since each individual femur has a different shape and size). Before we can compare these distributions and test how they depend on various regressors, we must first express each distribution on a common mesh. To this end, a canonical femur with 5580 vertices (step 5, red) is rotated, translated and nonrigidly deformed until it aligns with each individual femur (step 5, green). Once aligned, the surface data is mapped from the individual to the canonical femur and smoothed (step 6). The canonical surface mesh (which was constructed by averaging the shapes of several hundred individuals), and software to perform the registration, mapping and smoothing, are available for free download.^2^

Following registration, we used principal component analysis to build a point-based, statistical shape model from the *n* sets of canonical vertex coordinates obtained by applying the *n* nonrigid deformations. Let *X_j_* be the 16740-element vector formed by concatenating the canonical vertex coordinates following registration with individual *j*, and let $\hat{X}=\frac{1}{n}\Sigma_{j=1}^{n}X_{j}$. Then the principal modes of shape variation are the first *n* − 1 eigenvectors *m_i_* (*i* = 0… *n* − 2) of the sample covariance matrix $\frac{1}{n-1}\Sigma_{j=1}^{n}\left(X_{j}-\hat{X}\right){\left(X_{j}-\hat{X}\right)}^{T}$. Individual femurs may then be represented according to $X_{j}=\hat{X}+\Sigma_{i=0}^{n-2}S_{i}m_{i}$: in other words, the mean shape plus a certain amount of each shape mode. For each individual *j*, the shape coefficients *S_i_* are given by $S_{i}=\left(X_{j}-\hat{X}\right)\cdot m_{i}$.

**Fig. 2:**
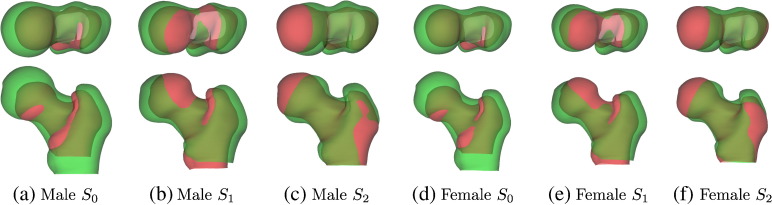
The first three modes of the statistical shape models, ± 3 standard deviations, accounting for 79% of the population variance in each cohort. Green is + 3 standard deviations, red is − 3 standard deviations.

The first three shape modes for the two cohorts are shown in Fig. 2. They are visualised by plotting $\hat{X}+S_{i}m_{i}$ with *S*_*i*_ = + 3 standard deviations (green) and − 3 standard deviations (red). *S*_0_ accounts for 58% of the shape variation observed in the males and 59% in the females. It captures typical, anisotropic size variation between individuals, with approximately 7% length change per standard deviation of *S*_0_ in the proximal–distal direction, and 4% per standard deviation in the other two directions. The next most dominant mode, *S*_1_, corresponds roughly to neck-shaft angle and accounts for 15% of the shape variation in the males and 14% in the females. *S*_2_ captures mostly changes in femoral neck axis length, accounting for 6% of the shape variation in both cohorts.

Finally, we used statistical parametric mapping (SPM) [5], as implemented in the SurfStat package [22], to fit a general linear model (GLM) to the *n* sets of registered data (Fig. 1, step 7), the aim being to explain the data at each vertex in terms of covariates of interest (e.g. *S*_0_) and also confounding covariates (e.g. age, scanning site). *F* or *t*-statistics can be calculated at each vertex, to test whether the data depends significantly on the covariates, with random field theory furnishing the corresponding *p*-values, corrected for multiple comparisons to control the overall image-wise chance of false positives (step 8). We initially fitted the GLM 1 + *S*_0_ + Age + *Σ*_*i* = 1_^5^*S*_*i*_ + Site to the CMSD data for the male and female groups separately, and then performed an *F*-test on *S*_0_, to test whether CMSD depends on femur size.^3^ In selecting this model, we anticipated age and scanning site to be confounding variables, and also allowed for nonrigid shape variation (*S*_1_… *S*_5_), which may have a genuine effect on CMSD and may also affect it through systematic misregistration [6]. We performed a limited amount of data exploration to arrive at this model, with implications for statistical inference, as discussed in Section 4. We subsequently fitted the same GLM to the CTh and CBMD data, again for the male and female groups separately, in order to measure how cortical thickness and cortical bone mineral density depend on *S*_0_ within regions of interest identified by the primary CMSD analysis.

## 3 Results

### 3.1 SPM

**Fig. 3:**
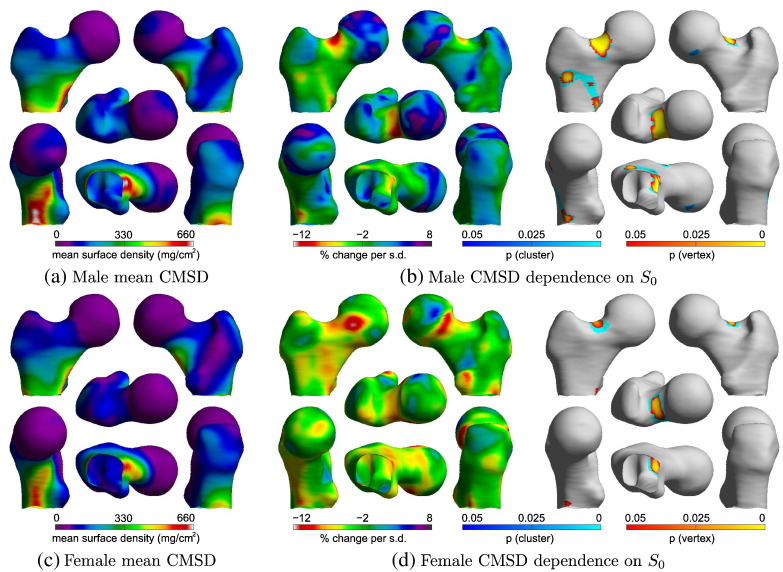
SPM analysis of the relationship between CMSD and femur size. The GLM fitted was 1 + *S*_0_ + Age + *Σ*_*i* = 1_^5^*S*_*i*_ + Site. The percentage change maps are derived from the *S*_0_ coefficient in the GLM: they show the percentage change in CMSD per standard deviation increase in *S*_0_. The corresponding *p*-maps are for *F*-tests on *S*_0_. The *p*-maps are based on the magnitudes of vertex peaks (yellow-orange colour map, sensitive to focal effects) and on the extent of connected clusters exceeding an uncorrected *p*-value threshold of 0.001 (cyan-blue colour map, sensitive to distributed effects).

Fig. 3 shows the results of the SPM analyses on the male and female cohorts. There is a region at the superior surface of the femoral neck where CMSD decreases with increased femur size for both males (*p* < 0.000005) and females (*p* < 0.001). In the male group, there is a second region, along the anterior intertrochanteric line, where CMSD also decreases with increased femur size. The different numbers, extents, and thus significances, of the male and female clusters may reflect different phenotypes, or may be attributed to the different sample sizes. We offer no further analysis of the intertrochanteric cluster in Fig. 3(b), since it is not validated in the female group and is less obviously associated with fracture than is the femoral neck cluster. We consider it an incidental finding that may, nevertheless, warrant further investigation in larger cohorts.

### 3.2 Magnitude and nature of the effect

For consistency and ease of comparison, we need to establish a specific region on the femoral neck for quantification of the *S*_0_ effect, and we choose for this purpose the male cluster in Fig. 3(b), which we henceforth refer to as the default femoral neck patch. Within this patch, Table 2 compares the CMSD effect with corresponding values for CTh and CBMD, derived by fitting the same GLM to the cortical thickness and cortical bone mineral density data. Males show a 6.87% decrease in CMSD per standard deviation increase in femur size, while for females the decrease is 6.74% per standard deviation. Coincidentally, one standard deviation of *S*_0_ corresponds to an approximately 7% change in linear size in the proximal–distal direction, so the effect amounts to an approximate 1% reduction in CMSD per 1% increase in proximal–distal size. As previously mentioned, CTh and CBMD estimates are less precise than those for CMSD, CTh slightly so, CBMD very significantly so [19]. Nevertheless, the values in Table 2 are strongly suggestive of an effect that is rooted in cortical thickness, with cortical bone mineral density playing a lesser role.

## 4 Discussion

Using the technique of cortical bone mapping, we have measured the CMSD, CTh and CBMD of the proximal femurs of 308 males and 125 females. We mapped the cortical measurements onto a canonical femur surface, and then fitted the GLM 1 + *S*_0_ + Age + *Σ*_*i* = 1_^5^*S*_*i*_ + Site to the CMSD, CTh and CBMD data for the male and female groups separately. Our principal finding was that CMSD decreased significantly with increased femur size *S*_0_ in a focal patch on the superior femoral neck. This finding was consistent across the male and female cohorts. It remains to explain how the GLM was chosen and to discuss the significance of the results.

### 4.1 Model selection

Table 3 discloses the full extent of the data exploration that led to the final statistical analysis. The a priori MrOS analysis plan, marked ^⁎^ in Table 3, was to investigate how the cortex depends on the subject's height, allowing for age, weight, shape and site. We anticipated a strong correlation between height and *S*_0_ — the actual correlation coefficient turned out to be 0.64 — and accordingly took care not to include both in the GLM, since SPM has no way of knowing which of any correlated regressors to attribute any shared variance to. We chose to model height, since it is the more convenient parameter to measure in practice.

The a priori analysis plan did indeed reveal a significant association between CMSD and height at the superior femoral neck, but post hoc data exploration revealed the true dependency to be with femur size, *S*_0_: compare the cluster extents and *p*-values in the first two rows of Table 3. Furthermore, while the model including weight explained the data very well, it revealed an unsurprising increase in CMSD with weight over almost all of the proximal femur. Heavier males tend to have larger femurs (correlation coefficient 0.42 in the MrOS cohort), so the highly significant effect in the second row of Table 3 needs careful interpretation. The 7.84% reduction in CMSD with *S*_0_ goes hand in hand with an increase in CMSD with weight, so it is difficult, with this particular model, to say whether larger bones do actually have reduced CMSD in the superior femoral cortex. That they do is revealed only in the final selected model, marked ^†^ in Table 3. This is a very clean model, with no significant correlations between the covariates, the largest correlation coefficient being − 0.24 between age and one of the site labels.

SPM *p*-maps are corrected for multiple comparisons over vertices, but not for multiple comparisons over different GLMs and contrasts. While data exploration is undoubtedly a valuable tool at the researcher's disposal, it must be accounted for when making claims of statistical significance, either by changing the test (e.g. Bonferroni correction, Scheffé's method) or by confirming the findings in an independent data set. The *S*_0_ effect in the selected model easily survives a conservative Bonferroni correction and is confirmed in the independent analysis of 125 females.

### 4.2 Femur size, functional adaptation and fracture risk

In Section 1, we noted a general consensus for the existence of a link between increased femur size (as measured by hip and femoral neck axis length in particular, and to a lesser extent femoral neck width as well) and increased fracture risk. There is also some evidence that cervical fractures are more strongly associated with femur size than are trochanteric fractures [7]. Rivadeneira et al. [16] observed a link between femoral neck width and fracture risk, and went as far as to suggest that “the only reason why a wider bone would not be stronger is if cortical dimensions were thinned to the point where bone strength is lost because of instability.” Our findings sit very comfortably alongside this existing body of work. We have previously observed a focal femoral neck defect in the contralateral hip of cervical fracture cases [14], and here we show how the defect is associated with increased femur size. Moreover, both finite element modelling and direct in vitro observation point to fracture initiation at the location of the defect, especially for tensile failures in stance, which tend to initiate at the mid-neck or head-neck junction [17]. Compressive failures in fall often initiate a little further laterally along the superior femoral neck, at the trochanteric fossa, although some have been observed at the head-neck junction too [17].

Our observations appear to be quite distinct from the well known phenomenon of age-related periosteal expansion, which also leads to cortical thinning at the femoral neck associated with an enlarged femur [2]. *S*_0_ and age were uncorrelated in our studies (correlation coefficients of − 0.018 in the males and 0.0031 in the females). It would seem, therefore, that we are observing a primary, spatial dependence of bone mass distribution on proximal femur geometry, rather than a secondary, temporal ageing effect. Because bone in the proximal femur is strongly influenced by functional adaptation to the prevalent loads, it is conceivable that focal osteoporosis of the superior femoral neck is a consequence of an individual's given femoral geometry, coupled with a lifetime of bone loss in stress-shielded regions [11].

Femoral size has only recently been tested in simulations of functional adaptation, albeit indirectly. Models developed by Machado et al. [10] predicted two opposing size effects: a marked decrease in femoral neck BMD with increasing femoral neck width, and a small increase in femoral neck BMD with increasing femoral neck length.^4^ Since the width effect was approximately an order of magnitude greater than the length effect, our observations are entirely consistent with this model. Further analysis of our results in Appendix A confirms that the femoral neck defect is indeed amplified in wide necks and attenuated in long necks. Machado et al. [10] also “verified that wider femoral necks present proportionally lesser BMD at the superolateral region of the neck comparatively to the inferomedial region”. All in all, there is a remarkable synergy between our observations and the functional adaptation models of Machado et al. [10].

There are other ways of understanding how mechanical adaptation of adult bone might be influenced by femur size. Following Lovejoy's interpretation of Frost's mechanostat [9], one could hypothesise that larger femurs have a greater amount of superior femoral neck bone tissue below the “trivial loading zone” that leads to bone removal though remodelling. Recent insights into bone adaptation through computer simulated dynamic mechanotransduction support this notion. Specifically, when micro-finite element models of the femur are subjected to walking simulations, the resultant femoral coronal sections show a startling similarity to true bone microstructure, with the bone tissue aligned along force trajectories at the expense of a large deficit at the superior femoral neck [8]. We hypothesise that this bone tissue deficit would be more extensive in larger femurs. Such biomechanically driven remodelling is believed to increase bending resistance while maintaining skeletal lightness [18]. Currey et al. [4] argue that functional adaptation of this nature is mainly beneficial in young adulthood during an individual's reproductive and most physically demanding years, well before fragility sets in.

The traditional explanation of the link between hip axis length and fracture risk is that larger bones create a greater bending moment in the femoral neck during a fall [7]. We suggest that the distribution of cortical bone at the superior femoral neck may also play an important role, at least when the neck is wide as well as long, as is generally the case. An important next step is to verify the association between bone size and the cortical defect in further cohorts covering different age and race/ethnic groups.

From a clinical perspective, while there are no practical interventions that might reduce the size of an individual's femur, a focal femoral neck defect can potentially be addressed through targeted exercise [1] or drugs [13], [15].

## 5 Conclusions

Traditional hip structure analysis is muddied by the interdependence of the various, intuitive measures used to characterize the geometry of the proximal femur. In this work, we have instead parameterized femoral size and shape along orthogonal vectors derived from principal component analysis of the population. Our main finding was a focal defect of the superior femoral neck associated with increased femur size. The defect appears to be consistent with models of functional adaptation, and may help explain previously observed links between femur size and fracture risk, as well as inform interventional strategies for reducing that risk.

## 6 Disclosures

KESP and GMT are the inventors on patent application WO2011/042738, which relates to cortical bone mapping.

## Figures and Tables

**Fig. 4:**
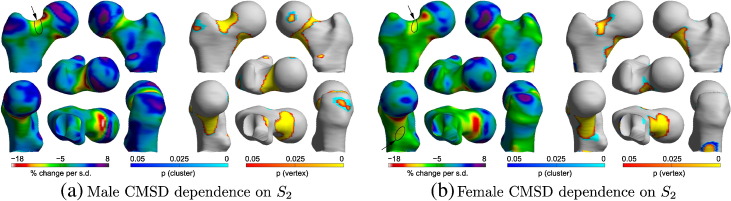
SPM analysis of the relationship between CMSD and *S*_2_. The GLM fitted was 1 + *S*_0_ + Age + *Σ*_*i* = 1_^5^*S*_*i*_ + Site. The percentage change maps are derived from the *S*_2_ coefficient in the GLM: they show the percentage change in CMSD per standard deviation increase in *S*_2_. The corresponding *p*-maps are for *F*-tests on *S*_2_. The arrows indicate regions where the effects cannot be attributed to systematic misregistration.

**Table 1:** Sample size, age, weight and height for the male and female cohorts. The values are given as mean ± standard deviation (range).

	n	Age (years)	Weight (kg)	Height (cm)
Males	308	73.5 ± 5.7 (65–91)	84.3 ± 14.0 (56–125)	174.3 ± 7.2 (147–198)
Females	125	76.8 ± 7.4 (53–98)	66.4 ± 11.1 (40–96)	158.1 ± 6.7 (141–175)

**Table 2:** Average percentage change in CMSD, CTh and CBMD per standard deviation increase in *S*_0_, within the default femoral neck patch.

	CMSD effect (% per s.d.)	CTh effect (% per s.d.)	CBMD effect (% per s.d.)
Males	− 6.87	− 6.74	+ 0.07
Females	− 6.74	− 4.90	− 1.86

**Table 3:** Characteristics of the femoral neck SPM cluster for various models and cohorts. The rightmost column quantifies the effect within the default femoral neck patch. The a priori analysis plan is marked ^⁎^, while the final selected model is marked ^†^.

	GLM for cortical mass surface density	Contrast	*p* (cluster)	Extent (vertices)	Effect (% per s.d.)
Exploration	1 + Hgt + Age + Wgt + Shp + Site^⁎^	Hgt^⁎^	2.20 × 10^− 2^	40	− 3.50
n = 308	1 + *S*_0_ + Age + Wgt + Shp + Site	*S*_0_	5.09 × 10^− 7^	208	− 7.84
Males	1 + *S*_0_ + Age + Shp + Site^†^	*S*_0_^†^	1.15 × 10^− 6^	195	− 6.87

Confirmation n = 125 females	1 + *S*_0_ + Age + Shp + Site^†^	*S*_0_^†^	8.51 × 10^− 4^	89	− 6.74

## Appendix A The effect of *S*_2_ on cortical mass surface density

The models of functional adaptation in [10] predict dramatically reduced femoral neck BMD with wider femoral necks, but slightly increased BMD with longer femoral necks. While this is consistent with our main finding for overall femur “size”, it does beg the question as to whether we can detect the opposing width-length effects in the CT data. We therefore undertook a tentative investigation of *S*_2_, which corresponds roughly to femoral neck axis length (see Fig. 2).

SPM analyses of dependencies on *S_i_* (*i* > 0) are challenging, because it is difficult to disentangle true effects from inevitable misregistration artefacts. Femurs with a large *S*_1_ tend to register with the canonical femur one way, those with a small *S*_1_ another way, and likewise with the other shape modes. Consequently, each individual's cortical distribution “slips” around the canonical surface in a manner that depends on shape, and the resulting artefactual variation may be incorrectly interpreted as a true effect [6].

Proceeding, then, with due caution, Fig. 4 suggests that CMSD might depend on *S*_2_ in both the male and female cohorts. A scale-comparison heuristic [6] reveals that most of the significant clusters can in fact be explained by systematic misregistration, apart from at the small regions indicated by arrows, where > 5 mm of misregistration per standard deviation of *S*_2_ would be required to account for the effect, which is somewhat implausible given our understanding of the registration algorithm. There does, therefore, appear to be a genuine dependence of CMSD on *S*_2_ at the superior femoral neck. Larger values of *S*_2_ (shorter, wider necks) are associated with less CMSD, whereas smaller values of *S*_2_ (longer, narrower necks) are associated with more CMSD. This is consistent with the modelling in [10].

## Footnotes

1 http://mi.eng.cam.ac.uk/rwp/stradwin

2 http://mi.eng.cam.ac.uk/ahg/wxRegSurf

3 For concision, and in common with many statistics packages, we use the model formula to specify the independent variables in the GLM. A model formula of the form 1 + *Σ*_*i* = 1_^5^*S*_*i*_ implies the GLM *y*_*j*_ = *β*_0,*j*_ + *Σ*_*i* = 1_^5^*β*_*i*,*j*_*S*_*i*_ + *ε*_*j*_, where *y_j_* is the dependent data (in this case, CMSD) at vertex *j*, *β*_*i*,*j*_ are the model coefficients and *ε_j_* is the residual error.

4 Machado et al. [10] also considered an adjusted model to account for increased loading on very long femoral necks, and this model predicted a greater increase in BMD with neck length. For an isotropically expanding femur, the combined width and length effects sum to a − 1.4%/+ 0.3% change in femoral neck BMD per 1% increase in linear size for the standard/very-long-neck models.
